# From Ion Current to Electroosmotic Flow Rectification in Asymmetric Nanopore Membranes

**DOI:** 10.3390/nano7120445

**Published:** 2017-12-14

**Authors:** Juliette Experton, Xiaojian Wu, Charles R. Martin

**Affiliations:** Department of Chemistry, University of Florida, Gainesville, FL 32611, USA; jexperton@ufl.edu (J.E.); xwu@chem.ufl.edu (X.W.)

**Keywords:** ion-current rectification, electroosmotic flow rectification, nanopores, diodes

## Abstract

Asymmetrically shaped nanopores have been shown to rectify the ionic current flowing through pores in a fashion similar to a p-n junction in a solid-state diode. Such asymmetric nanopores include conical pores in polymeric membranes and pyramidal pores in mica membranes. We review here both theoretical and experimental aspects of this ion current rectification phenomenon. A simple intuitive model for rectification, stemming from previously published more quantitative models, is discussed. We also review experimental results on controlling the extent and sign of rectification. It was shown that ion current rectification produces a related rectification of electroosmotic flow (EOF) through asymmetric pore membranes. We review results that show how to measure and modulate this EOF rectification phenomenon. Finally, EOF rectification led to the development of an electroosmotic pump that works under alternating current (AC), as opposed to the currently available direct current EOF pumps. Experimental results on AC EOF rectification are reviewed, and advantages of using AC to drive EOF are discussed.

## 1. Introduction

Nanofluidics involves the study and control of the transport of fluids through spatially confined nanostructures, such as nanopores [1,2,3]. Inside these nanostructures, the physical constraints due to charge, chemistry, or roughness of the surface can affect the behavior of the fluid [4]. Effects such as modified flow profile [5,6], permselectivity [7,8] and electrophoresis [9,10] have been observed and can potentially be used for analytical separation [9] or lab-on-a-chip applications [11,12]. In this study, we will focus on two nanofluidic rectification effects: ion current rectification (ICR) [13] and electroosmotic flow rectification (EFR) [14]. Both phenomena are observed when an electrolyte solution is confined in asymmetrically shaped nanopores within a film or membrane.

ICR was first described by Wei et al. in 1997 [15] and since then has been demonstrated inside conical nanopore membranes [16,17], glass nanopipettes [18,19], photolithographed nanotunnels [20], and pores with an asymmetric charge distribution pattern [21,22,23]. As will be discussed here, the asymmetric geometry causes the ionic resistance of the solution within the pore to be dependent on the direction of the current flowing through the pore [10,13]. In analogy with a solid-state diode, there is a forward voltage bias that leads to a low pore-resistivity state and high ionic current [24,25]. When current flows in the opposite direction (reverse voltage bias), the resistivity of the solution within the pore increases leading to a high resistivity state and lower ionic current through the pore.

Since the rate of electroosmotic flow (EOF) through the membrane is related to the resistivity of the solution [26], ICR yields a related EFR. EOF is induced by the motion of ions in the electrical double layer inside the nanopores [27,28] and is of considerable interest for pumping solution through nanofluidic devices [29,30]. EFR was first demonstrated using pyramidal pore mica membranes [31,32]. Later studies have shown EFR in conical pore polymeric membranes [33] and photolithographed nanotunnels [34].

In this review, we first describe the preparation of asymmetric nanopore membranes with a homogeneous surface charge distribution, focusing on two different types of membranes: mica and polyethylene terephthalate (PET). We then explain the origin and theory of ICR in asymmetric pore geometries and present a simple intuitive model for rectification. The effects of pore surface charge and chemistry on ICR are evaluated. Following these concepts, the theory of EFR is detailed along with the assessment of the effects of pore geometry, pore density, and current density. Finally, an application of EFR for a microfluidic pump under alternating current (AC) mode is presented.

Although this review focuses on solid-state nanopores, it is important to note that some biological pores have also shown ICR and EOF. Examples of such pores are the α-hemolysin [35,36,37], aerolysin [37], and fragaceatoxin C [38]. The experiments and theories reviewed here might be useful for understanding such phenomena in biological pores [39,40].

## 2. Preparation of Asymmetric Nanopore Membranes

**Figure 1:**
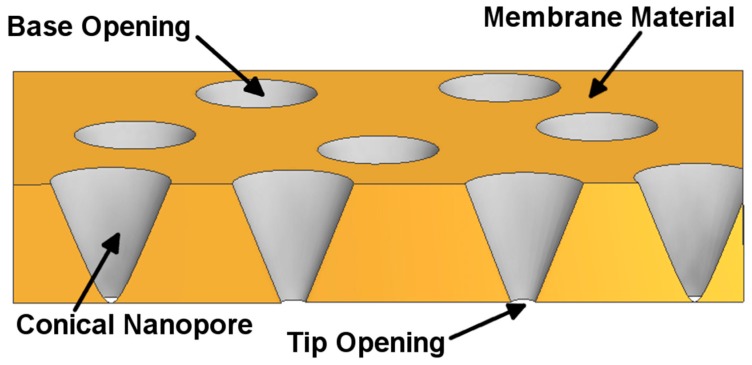
Schematic illustration of a membrane containing conical nanopores. Dimensions are not to scale.

An asymmetric nanopore membrane contains a single pore or multiple pores with a large opening (the base) on one face of the membrane and a small opening (the tip) on the opposite face (Figure 1). These nanopores can be pyramidal (such as those prepared in mica membranes [31,32]) or conical (such as those in polymeric [41,42,43,44] and glass membranes [14,18,45]). Typically, ICR has been observed with tip diameters less than 100 nm, depending on the chemistry of the pore and the electrolyte concentration [46,47,48,49,50]. Another condition for ICR measurement is that the nanopore should have excess surface charge [16,51]. The presence of anionic groups, such as silanate or carboxylate groups, on the surface of glass, mica, and polymeric nanopore membranes makes the pores negatively charged. Table 1 reviews characteristics of representative asymmetric nanopore systems.

**Figure 2:**
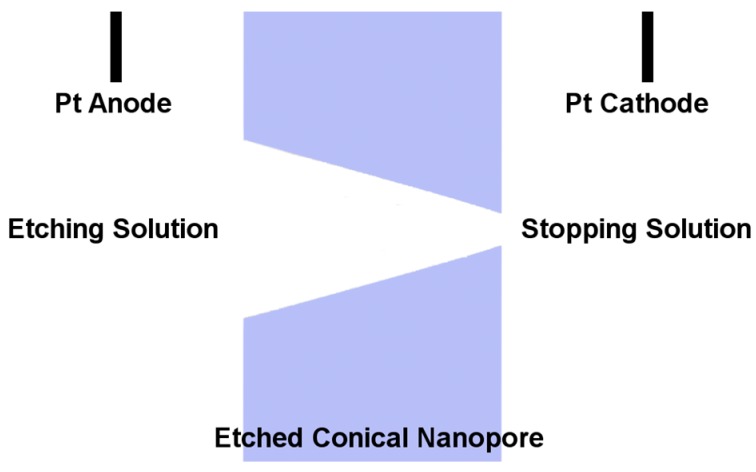
Schematic illustration of a conical nanopore during etching. Dimensions are not to scale.

Mica and polymeric asymmetric nanopore membranes were prepared using the anisotropic track-etching method (Figure 2) [59,60]. This method entails first irradiating mica or polymer films with energetic heavy ions, such as Au or Xe, yielding tracked films with a track density ranging from a single track to about 10^8^ tracks per square centimeter. A tracked film is then placed between two halves of a U-tube cell and the etching solution is added to one of the half cells (Figure 2). The other half cell is filled with a stopping solution to neutralize the etching solution transported through the nascent pore. For example, for a pyramidal mica nanopore membrane, the etching solution used was a 20% (*v*/*v*) HF solution, while the stopping solution was 10 M NaOH [31,32]. For conical nanopores in polyethylene terephthalate (PET) membranes, the etching solution was 9 M NaOH and the stopping solution was an acidic solution, commonly 1 M KCl and 1 M formic acid [33].

**Figure 3:**
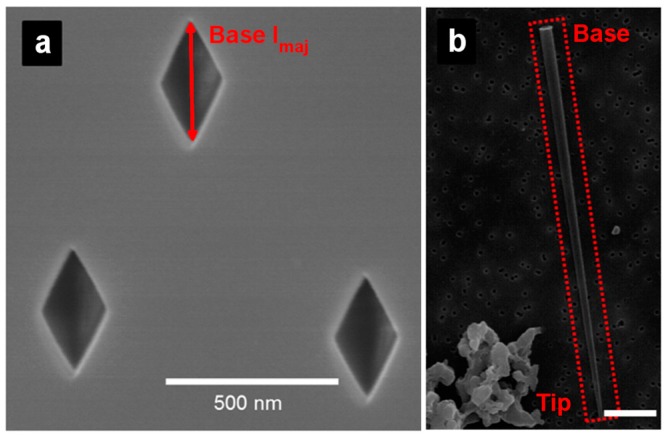
Scanning electron micrographs of (**a**) base openings in a track-etched pyramidal pore mica membrane and (**b**) a carbon replica of a pyramidal pore (scale bar: 3 µm) in the red dashed rectangle. The base major axis length *Base l*_maj_ is represented. Reproduced with permission from [32]. American Chemical Society, 2015.

**Figure 4:**
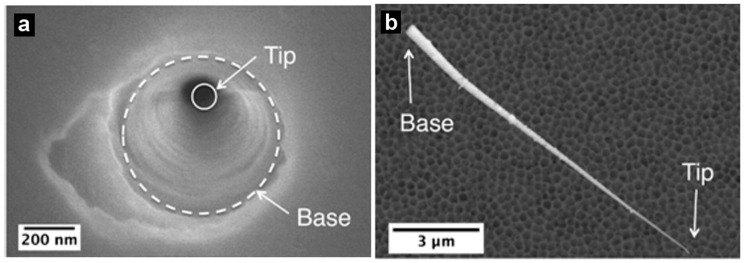
Scanning electron micrographs of (**a**) a base opening in a track-etched conical pore polyethylene terephthalate (PET) membrane and (**b**) a gold replica of a conical pore. Reproduced with permission from [33]. American Chemical Society, 2016.

During etching, a constant voltage is applied between two Pt or Au wires-placed to have the anode in the etching solution and the cathode in the stopping solution—and the current is measured (Figure 2). When the etching solution reaches the stopping solution (pore breakthrough), the current can flow through the membrane and the appearance of a current signal breakthrough is observed. The membrane can then be etched further in this anisotropic configuration or in a symmetric configuration with the etching solution in both of the half cells. The etching time is controlled to reach the desired tip and base sizes. Base diameters can be determined from electron micrographs, similar to that shown in Figure 3 and Figure 4 [32,33]. Commonly, the base diameter ranges from 0.1 to a few micrometers (Table 1) [46,47,48,49,50]. Tip diameters can be determined using an electrochemical method described previously [42,60,61].

Figure 3a shows an electron micrograph of the rhomboidal base in mica membranes [32]. The dimension of the base is described according to the base major axis length *Base l*_maj_, which was around 330 nm in this case. Similarly, the size of the tip is given by the tip major axis length *Tip l*_maj_, which was around 39 nm. A carbon replica of the pyramidal pore is shown in Figure 3b to visualize the entire pore.

Figure 4a presents an electron micrograph of a PET nanopore membrane with the base opening of the pore facing up, and the tip opening facing down [33]. The diameters of the tip and base were around 22 and 420 nm, respectively. A gold replica of the conical nanopore is also represented in Figure 4b.

## 3. Ion Current Rectification in Asymmetric Pores

**Figure 5:**
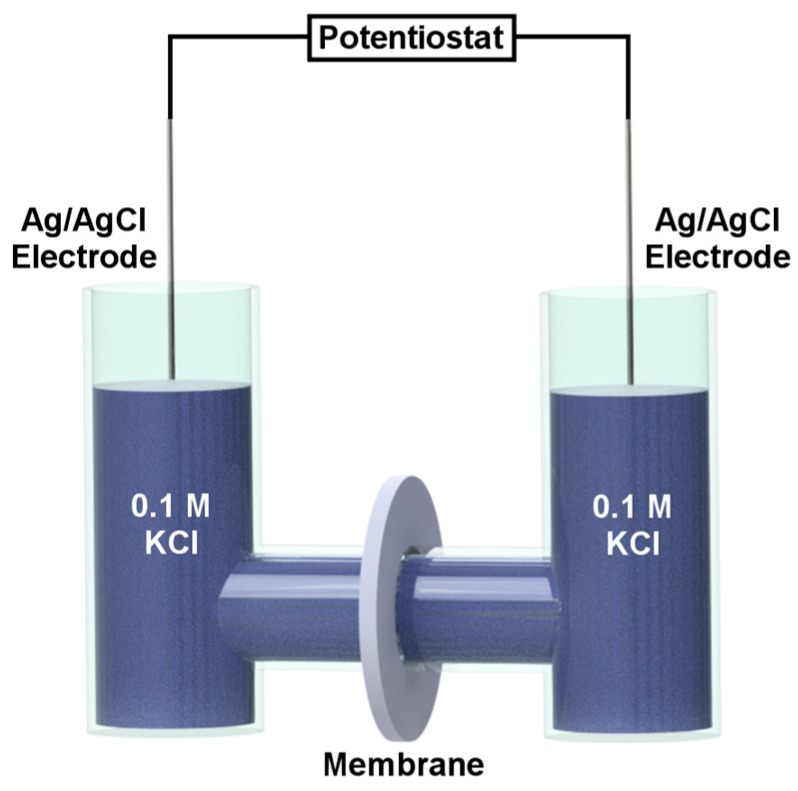
Schematic illustration of a U-tube cell used to measure current-voltage (I-V) curves associated with ion transport through the membrane. Dimensions are not to scale.

**Figure 6:**
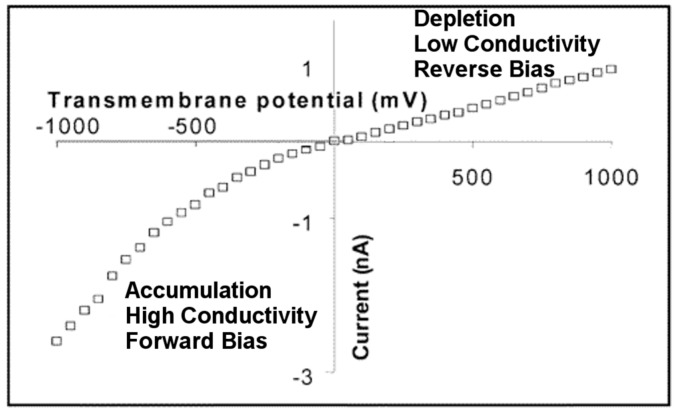
Rectified I-V curve in 0.1 M KCl measured across a PET membrane containing gold conical nanotubes. Reproduced with permission from [16]. American Chemical Society, 2004.

When an asymmetric (conical or pyramidal) nanopore membrane is placed between two half cells containing identical electrolyte solutions (for example, 0.1 M KCl), a current-voltage (I-V) curve associated with ion transport through the membrane can be measured using a reference electrode, such as Ag/AgCl, in each solution (Figure 5). The resulting ionic current is rectified; that is, at any absolute value of transmembrane voltage, the current is higher at negative voltages than at positive voltages (Figure 6) [16]. In analogy with a semiconductor p-n junction diode, there is a reverse bias at positive transmembrane voltages for which the current is low, and a forward bias at negative transmembrane voltages for which the current is high [62]. As we will discuss below, this is because at positive transmembrane voltages, the ionic resistivity is high inside the pores and the membrane is in the depletion state. At negative transmembrane voltages, the ionic resistivity is low inside the pores and the membrane is in the accumulation state.

The theory of ICR has been discussed in detail by others [13,18,63]. One interpretation assumes that rectification occurs in pores where the radius of the tip opening is comparable to the thickness of the electrical double layer at the charged pore walls [13,64]. Briefly, when the negatively charged pore surface comes in contact with an electrolyte solution, an electrical double layer forms at the pore wall [65]. One half of this double layer is comprised of the negative surface charge on the pore wall. The other half of the double layer consists of excess cations in the portion of the solution directly adjacent to the pore walls [66,67]. The excess cations are recruited from the bulk electrolyte solution to balance the negative surface charge on the pore. The thickness of this layer of excess cations, often called the Debye length *λ*_d_, depends on the ionic strength of the electrolyte solution (Equation (1)) [68]

(1) $$\lambda_{d}=\sqrt{\frac{\varepsilon_{r}\varepsilon_{0}k_{B}T}{\sum_{i}{\left(z_{i}e\right)}^{2}c_{i}}}$$

where *ε*_r_ is the dielectric constant of the solution, *ε*_0_ the permittivity of free space, *k*_B_ the Boltzmann constant, *T* the Kelvin temperature, *e* the elementary charge, *z*_i_ the ion valence, and *c*_i_ the concentration of the ion i. Equation (1) shows that the Debye length decreases with electrolyte concentration. For example, a 1 mM KCl solution in water at 25 °C has a Debye length of 9.6 nm; whereas at a concentration of 0.1 M, the Debye length is 0.96 nm.

**Figure 7:**
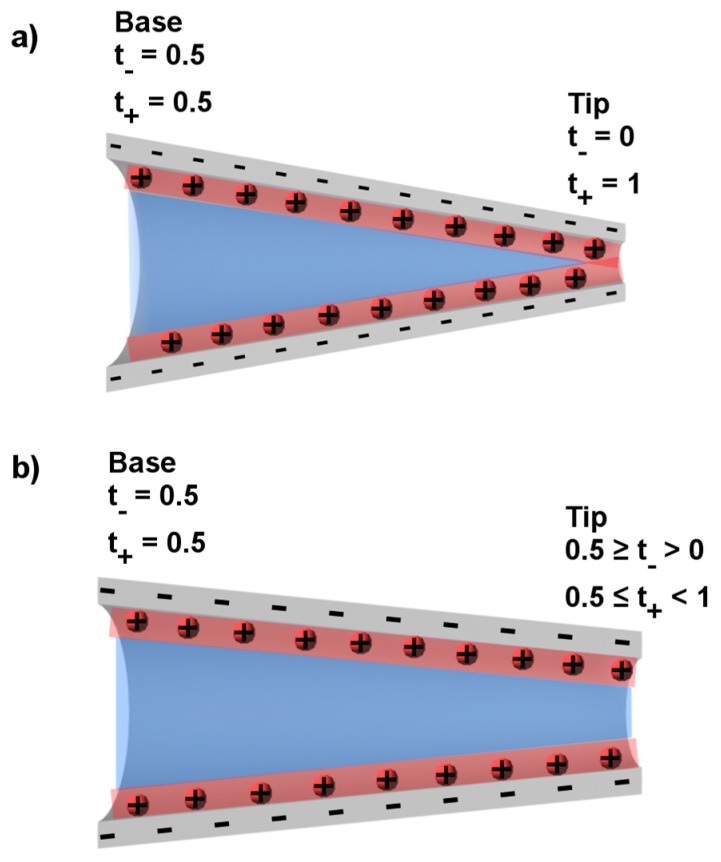
Schematic of conical pores (**a**) with the tip radius smaller or comparable to the double layer thickness and (**b**) with a tip radius larger than the double layer thickness. The double layer solution is represented by the red shading. The bulk solution is represented by the blue shading. Dimensions are not to scale.

If we assume for a particular pore and molarity of the electrolyte that the electrical double layer thickness at the pore wall, as given by *λ*_d_, is equivalent to the pore radius, then only double layer solution exists within the tip. This is shown in Figure 7a where the red shading represents the double layer along the pore wall, and the remaining solution, shaded in blue, is identical to the bulk electrolyte in contact with the pore. We see in Figure 7a that at the base, most of the solution within the pore is bulk solution but at the tip, in this example, all of the solution is double layer solution.

If this is true, then anions are excluded from the tip region [69,70], and when a current is passed through the pore, that current is carried through the tip only by the double layer cations. The tip region in this case is described as cation permselective [71]. The extent of cation permselectivity is measured in terms of transference numbers of cations and anions [72]. If the tip is ideally cation permselective (red shading in tip of Figure 7a), the transference number of cations *t*_+_ will be equal to 1, and the transference number of anions *t*_−_ will be equal to 0. At the base of the nanopore, the thickness of the double layer is negligible and does not influence the transport of ions. Therefore, the base region is non-permselective and, to a first approximation, *t*_+_ = *t*_−_ = 0.5 (Figure 7a).

In broadest terms, the pore is an ion current junction, where at one side of the junction *t*_+_ = 0.5 (the base side) and at the other side *t*_+_ = 1.0 (the tip side) (Figure 7a). When there is an ionic or electronic junction where *t*_+_ on one side of the junction is different from *t*_+_ on the other side, that junction will rectify the current flowing through the junction. We can now return to the analogy with a semi-conductor p-n junction, and note that it meets this criterion for rectification. This is because on the p-side of the junction, to a first approximation, *t*_+_ = 1.0, and on the n-side of the junction *t*_+_ = 0. As a result, voltage of one polarity drives charge carriers toward the junction, which results in charge accumulation and the high current forward bias case. The opposite polarity drives charge carriers away from the junction, resulting in charge depletion at the junction that yields the low current reverse bias case. The asymmetrical conical ion current junction (Figure 7a) does the same thing—one polarity (for a negatively charged pore cathode in the tip solution) results in depletion of charge carriers (cations and anions) from the tip region, and the opposite polarity results in accumulation of charge in the tip. This explains the asymmetry in the I-V curve observed above (Figure 6) [16], and has been demonstrated theoretically using finite element simulation [17,50,73].

This simple model for ICR assumed that the thickness of the electrical double layer was equivalent to the pore radius. This is sometimes called double layer overlap because, as indicated in Figure 7a, the double layers overlap in the tip region of the pore. However, if the pore radius is greater than the double layer thickness (Figure 7b), will the pore still rectify? Experimental data routinely show rectification in pores with tip radii as large as 25 nm [16,17,46] with some reports of rectification in pores with tip radii larger than 50 nm [31,47,74]. For all but the most dilute electrolyte solutions, these tip radii are larger than the thickness of the electrical double layer.

To understand these results, it is important to compare the number of moles of double layer cations that carry charge through the double layer region in the tip (shaded in red in Figure 7b) to the number of moles of ions (cations and anions) in the bulk solution in the tip (shaded in blue). If there is a significant number of moles of double layer cations compared to the moles of ions in the bulk of the tip, then *t*_+_ will be greater than 0.5 and the pore will rectify.

To illustrate this concept, the known anionic surface charge density on a PET surface (Table 1) [55,56] was converted to moles of carboxylate groups on the surface, and an equivalent number of moles of cations was assumed in the electrical double layer. An electrolyte solution of 10 mM KCl where the double layer thickness was 3 nm was considered. Then, assuming as per Figure 7b that the bulk solution in the tip is everything excluding the double layer, the number of moles of ions in that bulk solution part of the tip was calculated. The fraction of the total number of moles of ions that were double layer cations (moles of cations in the double layer divided by the sum of the moles of ions in the double layer and in the bulk solution) was then calculated. These results are expressed in Table 2 as the percent of ions in the double layer. These data were also used to calculate the cation transference number in the tip (Table 2). Details of these calculations are given in the supplementary material.

The first point to make about Table 2 is that total overlap (Figure 7a) would not be achieved for any of these pores because all of the radii are greater than the double layer thickness, 3 nm. Nevertheless, pores with tip radii of 10 nm or less have far greater moles of double layer cations in the tip than moles of bulk ions, and, in agreement with experimental data [17,47,75], such pores rectify. Returning to the discussion concerning the importance of the cation transference number in the tip on rectification, *t*_+_ of ~0.9 and above are obtained for the smallest tips (Table 2). However, it can also be noted that any junction will rectify where *t*_+_ on one side is not the same as *t*_+_ on the other. Assuming *t*_+_ = 0.5 in the base of the pore, Table 2 predicts that pores with tip radii as large as 100 nm will rectify in 10 mM KCl. These results are important because they confirm experimental data that show that the total overlap case (Figure 7a) is not required to achieve ICR.

**Figure 8:**
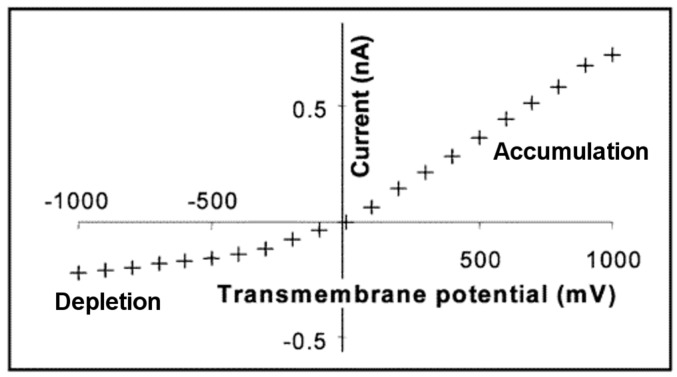
Rectified I-V curve in 0.1 M KF (pH 6.6) measured across a PET membrane containing gold conical nanotubes modified with mercaptoethylammonium cations. Reproduced with permission from [16]. American Chemical Society, 2004.

The final issue to discuss is the effect of the sign of the surface charge on ICR. This issue is experimentally problematic because the polymer and glass conical pores typically studied have a negative surface charge (Table 1). To obtain a system where the surface charge could be either positive or negative, Siwy et al. [16] coated the pore walls in conical pore polymeric membranes with corresponding conical gold nanotubes. Surface chemistry was then used to control the sign of the charge on the gold nanotube walls. For example, the gold nanotubes were made negatively charged simply by using KCl as the electrolyte, since it is well known that chloride ions adsorb to gold [76,77]. These nanotubes were shown to rectify in the expected way for a pore with negative surface charge (Figure 6). Siwy et al. then attached a cationic thiol, mercaptoethylammonium, to the nanotubes to make the surface charge positive. These nanopores rectified the current with an opposite polarity, as shown in Figure 8.

## 4. Electroosmotic Flow Rectification in Asymmetric Pores

As discussed above, asymmetric nanopore membranes show ICR because the ionic resistivity of the pore varies with the sign of the voltage difference applied across the membrane. One polarity results in depletion of electrolyte ions from the tip region and, therefore, high pore resistivity; the opposite polarity results in accumulation of ions in the tip and low pore resistivity. It was suggested that this change in pore resistivity with applied transmembrane voltage would yield a corresponding change in the EOF velocity *v*_eof_ (in mm s^−1^) through the membrane [31]. This can be understood through a consideration of the Helmholtz–Smoluchowski equation (Equation (2)) for the EOF velocity [27]

(2) $$\upsilon_{\mathrm{eof}}=-\frac{\varepsilon \zeta E\left(x\right)}{\eta }$$

where *ε* and *η* are the permittivity and viscosity of the solution within the pore, respectively, *E*(*x*) is the linear electric field gradient through the nanopore and *ζ* is the zeta potential of the pore wall. Equation (2) shows that *v*_eof_ increases with the electric field gradient *E(x)*.

In experiments where EOF is driven by a constant current density *J*_app_ [26,31,32], Equation (2) can be rearranged to

(3) $$\upsilon_{\mathrm{eof}}=-\frac{\varepsilon \zeta J_{\mathrm{app}}\rho }{\eta }$$

where *ρ* is the pore resistivity. Equation (3) shows that *v*_eof_ increases with *ρ*. Therefore, the high solution resistivity obtained when the pore is in the depletion state should yield a higher value of *v*_eof_ than when the pore is in the accumulation state. In agreement with this discussion, this is the experimentally observed result [31].

It is important to point out that both of these equations assume that the electrical double layer is much thinner than the pore radius [26]. This criterion is met for the membranes used for EOF rectification studies because the tip diameters were 17 nm or larger (Table 1), which is much larger than the Debye length for the electrolyte solutions used (<3 nm).

**Figure 9:**
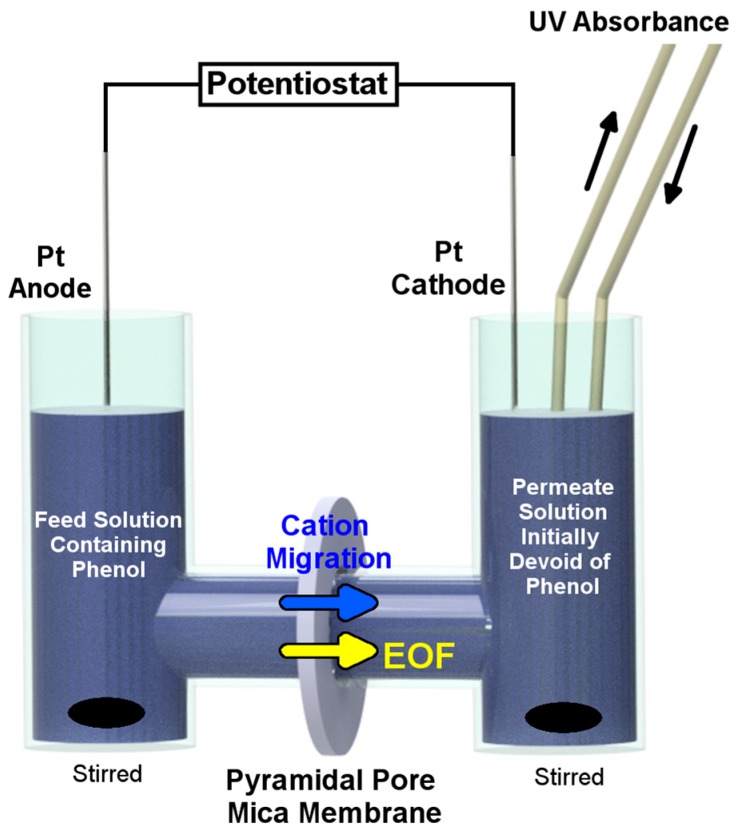
Schematic illustration of the permeation cell used to measure the flux of phenol (electroosmotic flow (EOF)) from the feed solution through the pyramidal pore mica membrane and to the receiver solution. The mica membrane was mounted with the base facing the feed solution to measure base-to-tip flux, or with the base facing the receiver solution to measure tip-to-base flux. Dimensions are not to scale.

To measure *v*_eof_, a mica membrane containing pyramidal nanopores was mounted in a simple permeation cell separating a feed and a permeate solution (Figure 9) [31,32]. A neutral chromophore, phenol, was transported from the feed to the permeate solution while a constant current of 100 µA was applied between Pt electrodes on either side of the membrane. The concentration of phenol in the permeate solution was measured with time and was used to calculate the flux of phenol and the corresponding EOF velocity [31,32].

**Figure 10:**
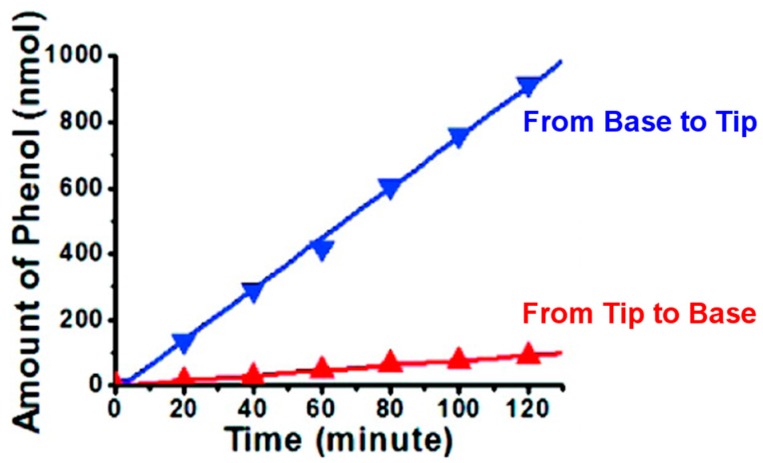
Fluxes of phenol from tip to base and from base to tip measured through a mica membrane containing pyramidal pores with tip and base lengths, *Tip* and *Base l*_maj_, of 17 and 122 nm, respectively. The feed solution initially contained 10 mM phosphate buffer (pH 7.0) and 10 mM phenol, and the permeate solution initially contained 10 mM phosphate buffer (pH 7.0). Reproduced with permission from [31]. American Chemical Society, 2010.

Figure 10 shows the resulting amount of phenol passing through the membrane as a function of time. The slopes of the lines represent the EOF fluxes from base to tip and from tip to base. In the blue curve, the cathode was facing the tip of the nanopores. Therefore, the pores were in the depletion state (high *ρ*, *vide supra*) and EOF was driven from base to tip. In the red curve, the cathode was facing the base. Therefore, the pores were in the accumulation state (low *ρ*) and EOF was driven from tip to base. These studies demonstrate EOF rectification because the flux is higher from base to tip than from tip to base.

The extent of ICR can be quantified by the ICR ratio *r*_ic_, which is the ratio, at any given absolute value of voltage, of the absolute values of current at positive and negative voltages [13]. The EFR ratio *r*_eof_ is calculated from the ratio of the velocities *v*_eof_ base-to-tip over tip-to-base. Table 3 shows *v*_eof_ from base to tip and tip to base, *r*_eof_ and *r*_ic_, for different tip and base lengths, *Tip l*_maj_ and *Base l*_maj_, of pyramidal pores in mica membranes [31]. A key result of these studies is that the two rectification ratios are linearly related.

Table 4 represents *r*_eof_ for different values of *J*_app_ and for two pore densities, 10^6^ and 10^7^ cm^−2^ [32]. For both pore densities, an increase in *r*_eof_ with *J*_app_ was observed. Equation (3) suggests that *v*_eof_ is proportional to *J*_app_ and *ρ* and *ζ*, which both vary with *J*_app_. It was shown that *ρ* and *ζ* are independent of *J*_app_ in the depletion state but decrease with *J*_app_ in the accumulation state. Therefore, *v*_eof_ from base to tip varied linearly with *J*_app_, and *v*_eof_ from tip to base varied non-linearly with *J*_app_. This explains why *r*_eof_ increases with *J*_app_. Additionally, *r*_eof_ was shown to be higher with lower pore densities. This is due to the higher electric field inside the pores in the accumulation state with the 10^7^ cm^−2^ pore density membranes. Therefore, *v*_eof_ from tip to base is higher with the 10^7^ cm^−2^ membranes.

## 5. Alternating Current Electroosmotic Flow Pump

The concept of EFR in a conical nanopore PET membrane was used to create a practical EOF pump [33]. Microfluidic devices and capillary electrophoresis columns cannot employ the common macroscale pump since high flow rates could damage the system, and the moving parts in these pumps are hard to scale down [78]. Therefore, there has been a considerable interest in using EOF pumps to operate such devices. The main disadvantage of EOF pumping under direct current (DC) mode is that large voltages are typically required [78]. These large voltages induce water electrolysis, which creates bubbles and changes the pH of the solution [78,79,80]. The EOF pump reviewed here minimizes the generation of water electrolysis since it operates under AC mode and low voltages (<4 *V*_rms_).

**Figure 11:**
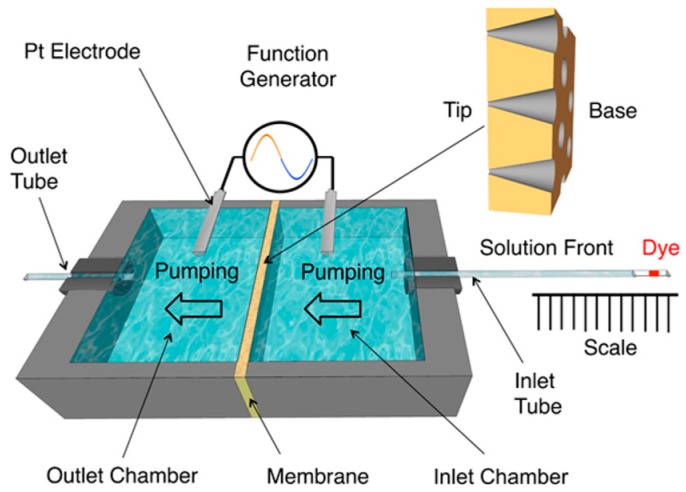
Schematic illustration of the alternating current (AC) EOF pump cell. A dye plug in the inlet tube was used to determine the EOF velocity. A net flow from base to tip (from right to left) was observed. Reproduced with permission from [33]. American Chemical Society, 2016.

As discussed above, the EOF is rectified in asymmetric nanopores because the EOF velocity from base to tip (depletion state) is higher than the EOF velocity from tip to base (accumulation state) [31,32]. Therefore, by applying a sinusoidal voltage waveform (AC current) across the membrane, a net flow from base to tip should be observed. This concept was proved experimentally and is reviewed here using the AC EOF pump cell represented schematically in Figure 11 [33]. A PET membrane containing conical pores with a tip diameter of ~22 nm was mounted between an inlet and an outlet chamber with the base side facing the inlet chamber. A sinusoidal voltage waveform was applied between two Pt electrodes placed in each chamber. The EOF rate was determined by measuring the displacement of a dye plug in a sealed inlet tube toward the inlet chamber.

**Figure 12:**
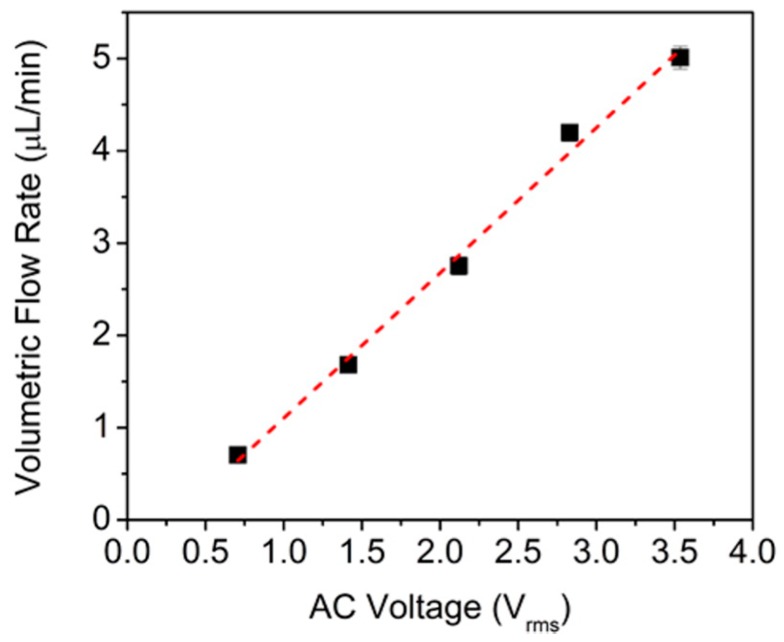
Volumetric flow rate as a function of the applied sinusoidal voltage. Reproduced with permission from [33]. American Chemical Society, 2016.

Knowing the displacement of the dye plug with time and the cross-section area of the inlet tube containing the dye plug, it was possible to calculate the net volumetric flow rate at five different values of amplitude of the applied sinusoidal voltage (Figure 12). The flow rate was found to increase linearly with AC voltage. As discussed previously, this is because the EOF velocity is proportional to the electric field inside the pores (Equation (2)).

**Figure 13:**
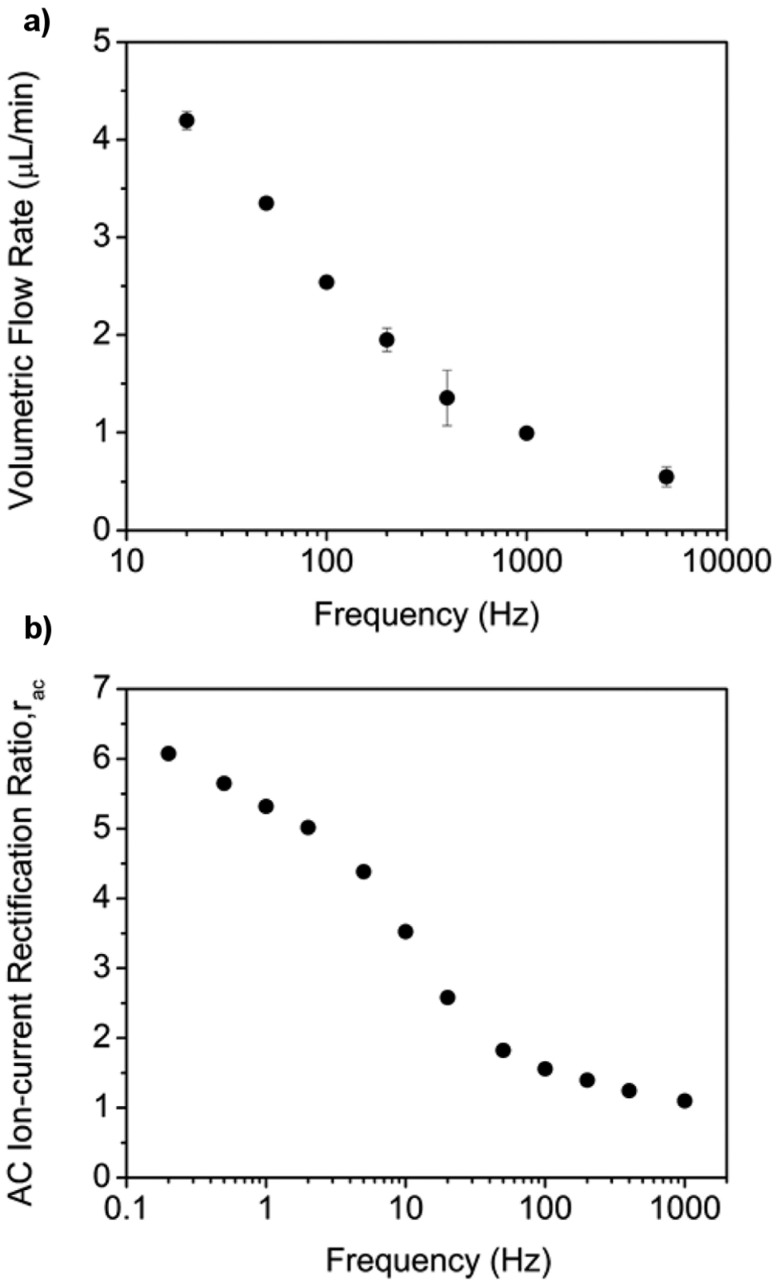
(**a**) Volumetric flow rate as a function of the frequency of the applied sinusoidal voltage with a magnitude of 2.8 *V*_rms_. (**b**) Ion current rectification (ICR) ratios as a function of the frequency of the applied sinusoidal voltage with a magnitude of 2.8 *V*_rms_. Reproduced with permission from [33]. American Chemical Society, 2016.

The frequency of the applied sinusoidal voltage was also shown to alter the extent of EFR and therefore the AC EOF pump flow rate [33]. As demonstrated in Figure 13a, flow rate decreases with increasing frequency. This is because, as suggested by previous studies [81,82,83,84], the ionic redistribution needed for accumulation and depletion cannot keep up with the voltage waveform at high frequencies. This was confirmed by measuring the AC ICR ratio *r*_ac_ as a function of frequency. As shown in Figure 13b, *r*_ac_ also decreases with increasing frequency.

The frequency range used for the EOF rate experiments (Figure 13a) was different than for the *r*_ac_ case (Figure 13b). As discussed in the original paper [33], gas evolution at the electrodes was observed at frequencies below about 20 Hz, and in the EOF studies, the gas bubbles generated interfered with the measurement of the flow rate. This is why the lowest frequency shown in Figure 13a is 20 Hz. Because flow is not involved in the measurement of *r*_ac_, lower frequency data could be obtained (Figure 13b). The highest frequency used in the *r*_ac_ experiments was lower than in the EOF rate case. This was caused by an instrumentation-based limitation on the rate at which the AC current could be sampled.

## 6. Conclusions

As reviewed here, asymmetric nanopore membranes are unique in that they induce a rectification of current and EOF when applying voltages of equal magnitude but opposite polarity across them. The extent of these rectification phenomena was shown to depend strongly on the pore chemistry, pore geometry, voltage or current applied across the membrane, and frequency. This ability to control the rectification ratio of the membrane is of great interest for applications such as the AC EOF pump described here. We reviewed the relation between the rectification ratio and the volumetric flow rate, which allowed us to regulate the pump. One aspect for future study would be to use an analyte ion to modify the quantity and sign of the pore surface charge. If this can be accomplished, a chemoresponsive EOF pump might be possible where the pump turns on and off in the presence of a specific ionic analyte.

## Figures and Tables

**Table 1:** Representative asymmetric nanopore systems.

Material	Surface Charge (mC·m^−2^)	Typical Base Diameter (µm)	Typical Tip Diameter (nm)	Electrolyte Solutions Used	References
Glass or Quartz	−0.5 [52]	25–100	20–150	0.1 mM–0.1 M KCl	[14,15,45]
Gold	−2 [53]	0.6	10	0.1 M KCl or KF	[16]
Mica	−340 [54]	0.1–0.5	10–50	10 mM Na_2_HPO_4_	[31,32]
polyethylene terephthalate (PET)	−12 [55,56]	0.1–0.6	2–30	0.001–1 M KCl	[17,33,47,57]
Polycarbonate	−2 [58]	0.1–3.5	50–100	/	[41,42]

**Table 2:** Calculations of the fractions of the total number of moles of ions that are double layer cations and of the transference numbers at the tip of different pore tip radii.

Tip Radius (nm)	Percent of Ions in the Double Layer	*t*_+_
5	94	0.97
10	72	0.85
20	46	0.72
50	22	0.59
100	12	0.54

**Table 3:** Electroosmotic flow (EOF) velocities, *v*_eof_, and rectification ratios of ionic current and EOF, *r*_ic_ and *r*_eof_, respectively, for five membranes with different tip and base major axis lengths, *Tip l*_maj_ and *Base l*_maj_. Reproduced with permission from [31]. American Chemical Society, 2010.

*Tip l*_maj_ (nm)	*Base l*_maj_ (nm)	*v*_eof_ (mm s^−1^)		*r*_eof_	*r*_ic_
		Base to Tip	Tip to Base		
17	122	3.8	0.37	10.3	5.3
35	244	1.7	0.35	4.9	2.7
52	366	0.55	0.32	1.7	1.3
70	488	0.32	0.23	1.4	1.2
11	11	12	12	1.0	1.0

**Table 4:** EOF rectification ratios for membranes with 10^6^ and 10^7^ pores cm^−2^ at different applied current densities *J*_app_. Reproduced with permission [32]. Copyright 2015, American Chemical Society.

*J*_app_^a^ (A cm^−2^)	*r*_eof_ 10^6^ Pores cm^−2^ Membrane	*r*_eof_ 10^7^ Pores cm^−2^ Membrane
0.22	-	1.8 ± 0.9
0.44	-	1.3 ± 0.4
1.11	4.5 ± 0.8	2.8 ± 0.7
2.21	5.8 ± 0.9	4.7 ± 0.9
3.32	9 ± 1	6 ± 1
4.43	12 ± 1	6 ± 1
5.54	12 ± 1	-

^a^ The current density was calculated based on the geometric mean of the base and tip openings of the pore.

## Supplementary Materials

Supplementary materials can be found at www.mdpi.com/2079-4991/7/12/445/s1.
