# Supplementary material for: From Ion Current to Electroosmotic Flow Rectification in Asymmetric Nanopore Membranes
Source: Nanomaterials (Basel). 2017 Dec 14;7(12):445. doi: 10.3390/nano7120445 (PMC5746935; doi:10.3390/nano7120445)
Supplement: Supplementary file 1 [file nanomaterials-07-00445-s001.pdf]

Supplementary Material.

From Ion Current to Electroosmotic Flow Rectification in Asymmetric Nanopore Membranes

*Juliette Experton, Xiaojian Wu and Charles R. Martin**

Department of Chemistry, University of Florida, Gainesville, Florida 32611, United States

*Correspondence: crmartin@chem.ufl.edu

Calculations of Percent of Ions in the Double Layer and Transference Number

To simplify our calculations, we assume that the tip is a cylinder of length h and radius r. The tip walls have a surface charge density of -12 mC m^{-2}, based on previously reported values for polyethylene terephthalate (PET) [1,2]. Inside the tip, there is a solution of 10 mM KCl which gives a thickness of the electrical double layer (Debye length) of 3 nm at 25 °C.

The surface charge density on the pore tip walls can be converted to a number of moles of carboxylate groups at the surface of the cylinder, n_{coo}, according to Equation S1, where F is the Faraday constant.

$$n_{COO} = \frac{1.2\times10^{-6}\ \times 2\pi rh}{F} \qquad \text{Equation S1}$$

If we assume an equivalent number of moles of cations (K^+) in the electrical double layer, n_{dl} (Equation S2).

$$n_{dl} = \ n_{COO}\ = \frac{1.2\times10^{-6}\ \times 2\pi rh}{F} \qquad \text{Equation S2}$$

To a first approximation, the number of moles of ions, n_b, which is the sum of the numbers of moles of K^+ and Cl^-, $n_{K,b}$ and $n_{Cl,b}$ respectively, in the bulk solution in the pore tip is determined by the bulk concentration (10 mM) (Equation S3).

$$n_b = \ n_{K,b} + n_{Cl,b} = 2 \times 10^{-5} \times \pi(r - 3 \times 10^{-7})^2 h \qquad \text{Equation S3}$$

Therefore the fraction of total number of moles of ions that are double layer cations, as a function of *r*, the radius of the pore tip in nm, is

$$\%_{dl} = \frac{n_{dl}}{n_b + d_{dl}} = \frac{0.12\, r}{0.12\, r + F \times 10^{-7}(r-3)^2} \times 100$$ Equation S4

The transference number of cations (K^+) through the tip, t_+, is defined as [3]

$$t_+ = \frac{(n_{dl} + n_{K,b}) \times u_{Na}}{(n_{dl} + n_{K,b}) \times u_K + n_{Cl,b} \times u_{Cl}}$$ Equation S5

Where u_K and u_{Cl} are the mobilities of K^+ and Cl^-, respectively.

Considering $u_K = 7.619 \times 10^{-4}\ cm^2\ s^{-1}\ V^{-1}$ and $u_{Cl} = 7.912 \times 10^{-4}\ cm^2\ s^{-1}\ V^{-1}$ [3],

$$t_+ = \frac{(0.24\, r + F \times 10^{-7}(r-3)^2) \times 7.619}{1.8286\, r + 15.824\, F \times 10^{-7}(r-3)^2}$$ Equation S6

References

1. Dejardin, P.; Vasina, E.N.; Berezkin, V.V.; Sobolev, V.D.; Volkov, V.I. Streaming potential in cylindrical pores of poly(ethylene terephthalate) track-etched membranes: Variation of apparent zeta potential with pore radius. *Langmuir* **2005**, *21*, 4680-4685.
2. Xue, J.M.; Xie, Y.B.; Yan, Y.; Ke, J.; Wang, Y.G. Surface charge density of the track-etched nanopores in polyethylene terephthalate foils. *Biomicrofluidics* **2009**, *3*, 8.
3. Bard, A.J.; Faulkner, L.R. Electrochemical methods: Fundamentals and applications, 2nd ed. John Wiley & Sons: New York, 2001; pp 65-69.
